# Dynamic expression of HOPX in alveolar epithelial cells reflects injury and repair during the progression of pulmonary fibrosis

**DOI:** 10.1038/s41598-018-31214-x

**Published:** 2018-08-28

**Authors:** Chiharu Ota, John-Poul Ng-Blichfeldt, Martina Korfei, Hani N. Alsafadi, Mareike Lehmann, Wioletta Skronska-Wasek, Martina M. De Santis, Andreas Guenther, Darcy E. Wagner, Melanie Königshoff

**Affiliations:** 1 Research Unit Lung Repair and Regeneration, Helmholtz Zentrum München and University Hospital of the Ludwig Maximilians Universität, Member of the German Center for Lung Research (DZL), Munich, Germany; 2 Department of Pediatrics, Tohoku University School of Medicine, Sendai, Japan; 3 Department of Molecular Pharmacology, Groningen Research Institute for Asthma and COPD (GRIAC), University of Groningen, Groningen, The Netherlands; 4 Department of Pulmonary and Critical Care, Justus-Liebig University School of Medicine, Member of the German Center for Lung Research (DZL), Giessen, Germany; 5 Department of Pulmonary and Critical Care, Justus-Liebig University School of Medicine, Agaplesion Lung Clinic Waldhof Elgershausen, Member of the German Center for Lung Research (DZL), Giessen, Germany; 6 Department of Experimental Medical Sciences, Lund Stem Cell Center, and Wallenberg Molecular Medicine Center, Lund University, Lund, Sweden; 7 Division of Pulmonary Sciences and Critical Care Medicine, Department of Medicine, University of Colorado, Denver, Aurora, CO USA

## Abstract

Mechanisms of injury and repair in alveolar epithelial cells (AECs) are critically involved in the progression of various lung diseases including idiopathic pulmonary fibrosis (IPF). Homeobox only protein x (HOPX) contributes to the formation of distal lung during development. In adult lung, alveolar epithelial type (AT) I cells express HOPX and lineage-labeled *Hopx*+ cells give rise to both ATI and ATII cells after pneumonectomy. However, the cell function of HOPX-expressing cells in adult fibrotic lung diseases has not been investigated. In this study, we have established a flow cytometry-based method to evaluate HOPX-expressing cells in the lung. HOPX expression in cultured ATII cells increased over culture time, which was accompanied by a decrease of proSP-C, an ATII marker. Moreover, HOPX expression was increased in AECs from bleomycin-instilled mouse lungs *in vivo*. Small interfering RNA-based knockdown of *Hopx* resulted in suppressing ATII-ATI trans-differentiation and activating cellular proliferation *in vitro*. In IPF lungs, HOPX expression was decreased in whole lungs and significantly correlated to a decline in lung function and progression of IPF. In conclusion, HOPX is upregulated during early alveolar injury and repair process in the lung. Decreased HOPX expression might contribute to failed regenerative processes in end-stage IPF lungs.

## Introduction

The lung is a complex organ which is directly exposed to numerous external stimuli, such as cigarette smoke, pathogens, air pollution, or particles from the environment^1^. The distal lung is composed of alveoli which are covered by alveolar epithelial type (AT) I cells and ATII cells. ATI cells are squamous, large, and flat cells, which cover 95% of the internal surface area of the alveoli^2^. ATI cells play an indispensable role for gas exchange and alveolar-capillary barrier function^2^. Thus, it is critically important to preserve and regenerate ATI cells for proper respiratory function. ATII cells maintain alveolar homeostasis as a “caretaker” of alveoli^3^ to produce and secrete surfactant proteins^4^, to keep the fluid balance of the alveoli^5^, and to serve as progenitor cells for ATI/ATII cells during lung turnover and repair^6^. Mechanisms of alveolar epithelial injury and repair have been proposed to be critically involved in the progression of various lung diseases, including idiopathic pulmonary fibrosis (IPF), a fatal interstitial lung disease^1^.

Homeobox only protein x (HOPX) is a Homeobox protein important for anterior-posterior patterning during organ development^7,8^. In the developing lung, HOPX contributes to the formation of distal lung^9^. In adult murine lung, ATI cells express HOPX^10^ and lineage-labeled *Hopx*+ cells, which are further positive for ATI cell markers, give rise to both ATI and ATII cells after pneumonectomy^11^. Thus, HOPX might be involved in the bidirectional trans-differentiation of ATII to ATI or ATI to ATII during adult alveolar repair. It is also known that HOPX-expressing cells serve as stem cells in intestinal epithelium^12^ or hair follicle^13^. Furthermore, HOPX is further described as a tumor suppressor gene in the lung that regulates cellular proliferation and metastasis^14,15^. However, the function of HOPX-expressing cells in the adult fibrotic lung has not yet been clarified. In the present study, we aimed to gain a detailed insight into the dynamics of HOPX-expressing alveolar epithelial cells (AECs) during lung injury and repair and established a flow cytometry (FCM)-based method to quantify the HOPX-expressing cells in AECs. We then investigated HOPX expression in an ATII-ATI trans-differentiation culture model *in vitro*, in bleomycin-induced mouse model of pulmonary fibrosis *in vivo*, and by database analyses of expression profiles of IPF lungs *in silico*.

## Results

### ProSP-C and HOPX expression during ATII-ATI trans-differentiation

**Figure 1:**
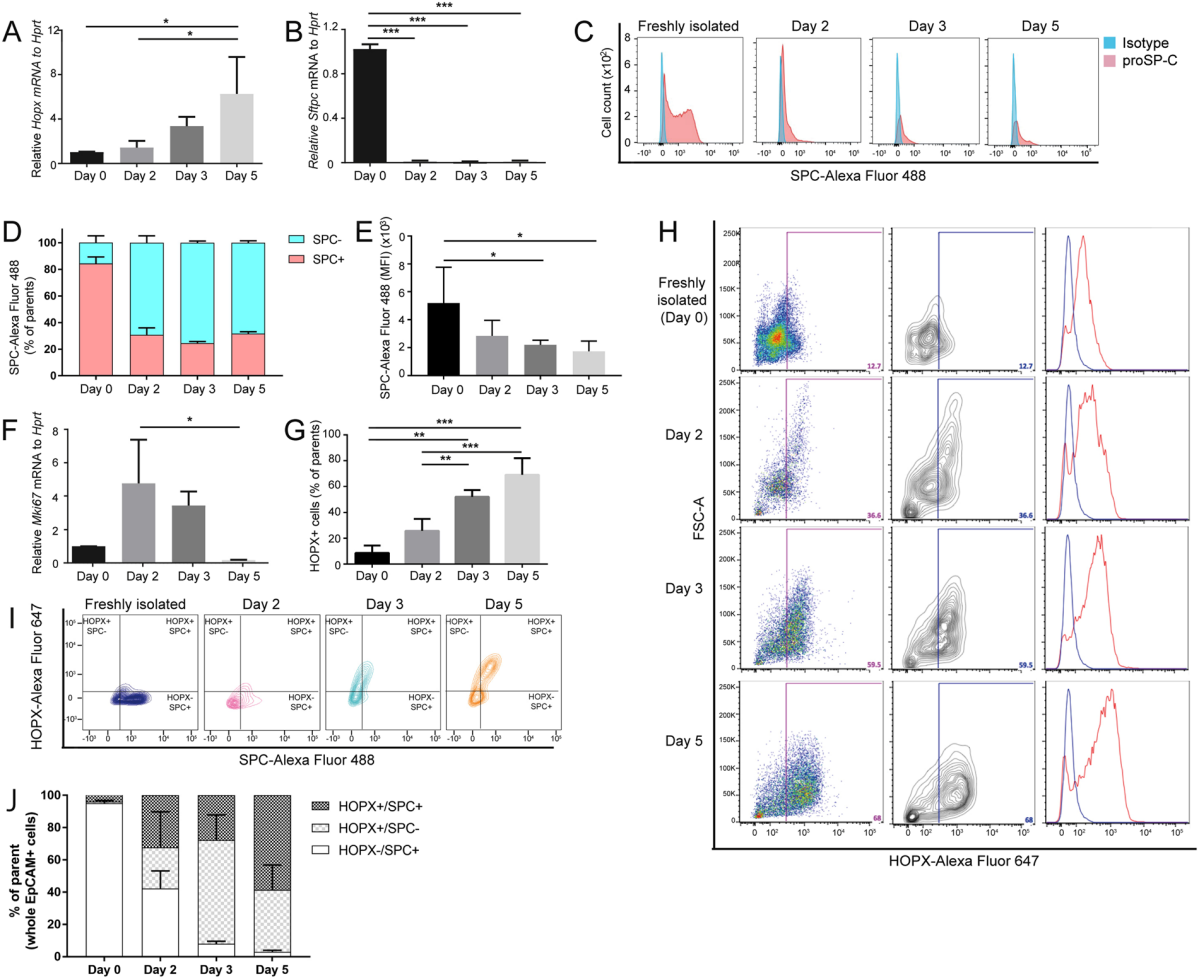
Expression of HOPX and proSP-C during ATII-ATI cell trans-differentiation *in vitro*. Quantitative (q) RT-PCR analysis of (**A**) *Hopx* (**B**) *Sftpc*, (**C**,**D**) FCM analysis of proSP-C expression over culture, (**E**) mean fluorescent intensity (MFI) of proSP-C-Alexa-fluor 488, and (**F**) qRT-PCR analysis of *Mki67* during 5 days culture of pmATII cells (n = 3). (**G**,**H**) FCM-based quantification of HOPX expression during the culture. (**I**) and (**J**) FCM-based quantification of HOPX/proSP-C co-expression during the culture (n = 3). *p < 0.05, **p < 0.01, ***p < 0.005.

To investigate the potential role of HOPX in adult lung injury/repair, we first investigated the changes of HOPX expression in primary mouse (pm) ATII cells (95+/−3% of proSP-C expression) from wildtype mice during 2D-culture *in vitro*. This *in vitro* culture system is well-established to study trans-differentiation of ATII cells into ATI-like cells^16–18^, a process known to be implicated in lung repair. *Hopx* expression was increased (Fig. 1A) over 5 days of culture along with decreased *Sftpc* expression, as evaluated by qRT-PCR (Fig. 1B) and FCM (Fig. 1C and D, mean fluorescent intensity (MFI) for Fig. 1E), which is consistent with previous reports^10,16^. Moreover, after an initial increase, the proliferation marker *Mki67* was decreased during the time course (Fig. 1F). We next optimized the FCM-based quantification of HOPX expression in pmATII cells using the same trans-differentiation system (Fig. 1G,H). HOPX expression was gradually increased during the culture (Fig. 1H). In addition, we evaluated the co-expression of proSP-C and HOPX within freshly isolated pmATII cells over culture time (Fig. 1I). We found that HOPX^+^/proSP-C^+^ cells as well as HOPX^+^/proSP-C^−^ cells were increased while HOPX^−^/proSP-C^+^ cells were decreased during trans-differentiation (Fig. 1J).

### HOPX expression was increased in the alveolar epithelium in bleomycin (BLM)-instilled lungs

**Figure 2:**
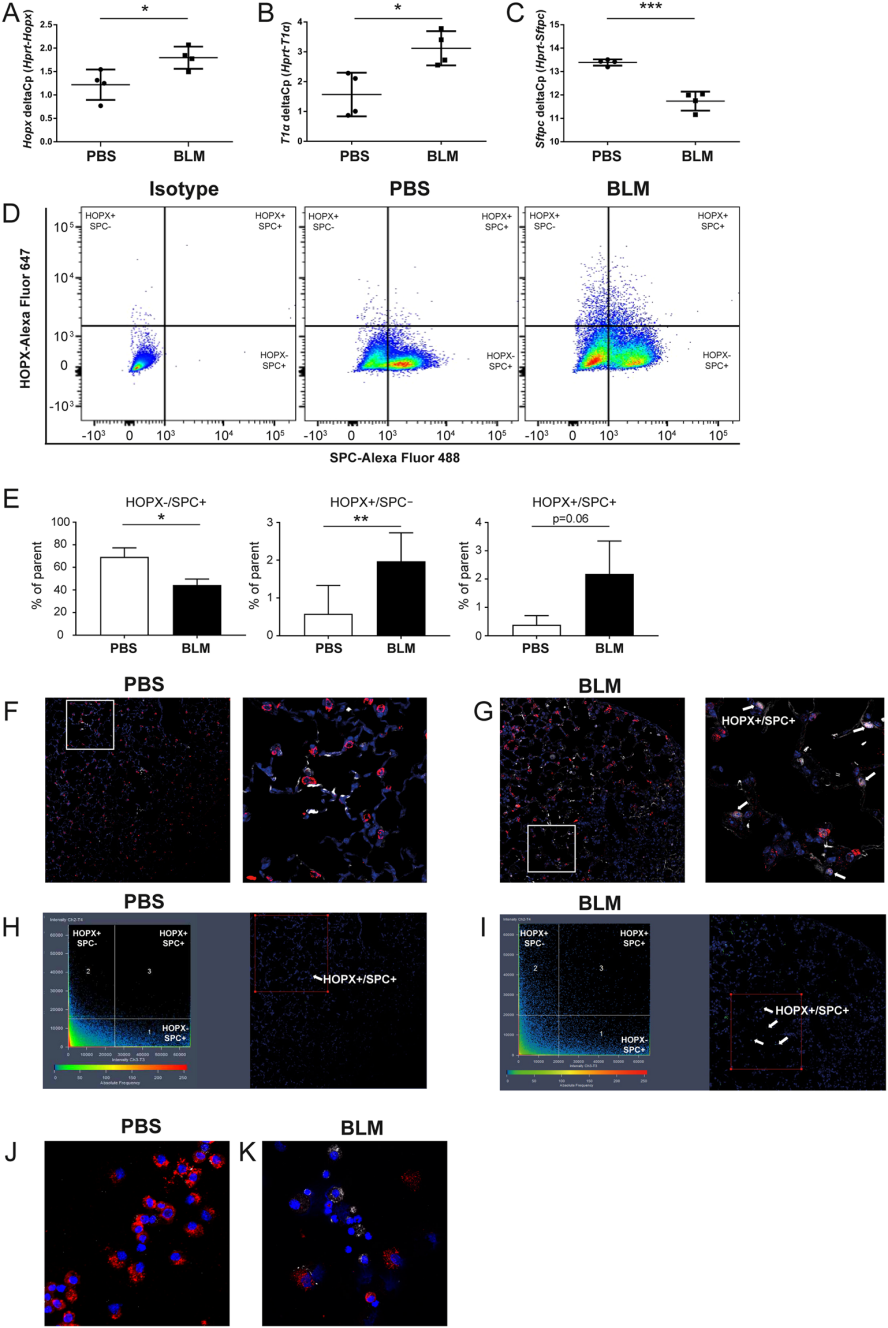
Expression of HOPX/proSP-C lung epithelial cell subpopulations in BLM-induced pulmonary fibrosis model *in vivo*. Quantitative RT-PCR analysis of (**A**) *Hopx* (**B**) *T1α*, and (**C**) *Sftpc* in EpCAM+ cells from PBS or BLM-instilled lungs (n = 4). (**D**) FCM-based evaluation of HOPX/proSP-C expression in pmATII cells from PBS or BLM-instilled lungs (representative images from n = 3). Quantification of (**E**) HOPX−/proSP-C+, HOPX+/proSP-C−, and HOPX+/proSP-C+ cells in PBS and BLM-instilled lungs (n = 3). Immunofluorescence staining (IF) of HOPX (white) and proSP-C (red) in the sections from (**F**) PBS and (**G**) BLM-instilled lungs. Visualization of the co-expression of HOPX and proSP-C from (**H**) PBS and (**I**) BLM-instilled lungs using ZEN2009 software. IF of HOPX (white) and proSP-C (red) in cytospun EpCAM+ cells from (**J**) PBS and (**K**) BLM-instilled lungs. *p < 0.05, **p < 0.01, ***p < 0.005.

Disturbed ATII to ATI cell trans-differentiation has been linked to lung fibrosis^16,17^. Thus, we next sought to investigate the expression changes of HOPX in fibrotic lung diseases using the FCM analysis. We evaluated *Hopx* expression in the mouse model of pulmonary fibrosis induced by intra-tracheal BLM instillation. We isolated ATII cells from phosphate buffered saline (PBS)-instilled lungs (PBS-pmATII cells) as a control group and BLM-instilled mouse lungs (BLM-pmATII cells) after 14 days of the initial PBS/BLM instillation. We found that mRNA expression of *Hopx* (Fig. 2A) and *T1α* (Fig. 2B) was significantly upregulated whereas *Sftpc* (Fig. 2C) was significantly downregulated in BLM-pmATII cells compared with PBS-pmATII cells. Next, we evaluated the expression of proSP-C and HOPX by FCM (Fig. 2D). Quantification of the FCM analysis revealed a significant decrease of HOPX^−^/proSP-C^+^ cells while HOPX^+^/proSP-C^+^ cells were significantly increased in BLM-pmATII cells compared to in PBS-pmATII cells (Fig. 2E). Importantly, further immunofluorescence (IF) also revealed that HOPX expression was increased in BLM-instilled lungs compared with PBS-instilled lungs *in situ* (Fig. 2F and G). The cells which co-expressed both HOPX and proSP-C were increased in BLM-lungs (Fig. 2G, shown in white arrows, and Fig. 2I, shown in green dots) compared to PBS-lungs (Fig. 2F and H). The co-expression of proSP-C and HOPX was also confirmed by IF of cytospun pmATIIs which were freshly isolated from PBS- (Fig. 2J) or BLM-instilled lungs (Fig. 2K).

### HOPX knockdown activated cellular proliferation

**Figure 3:**
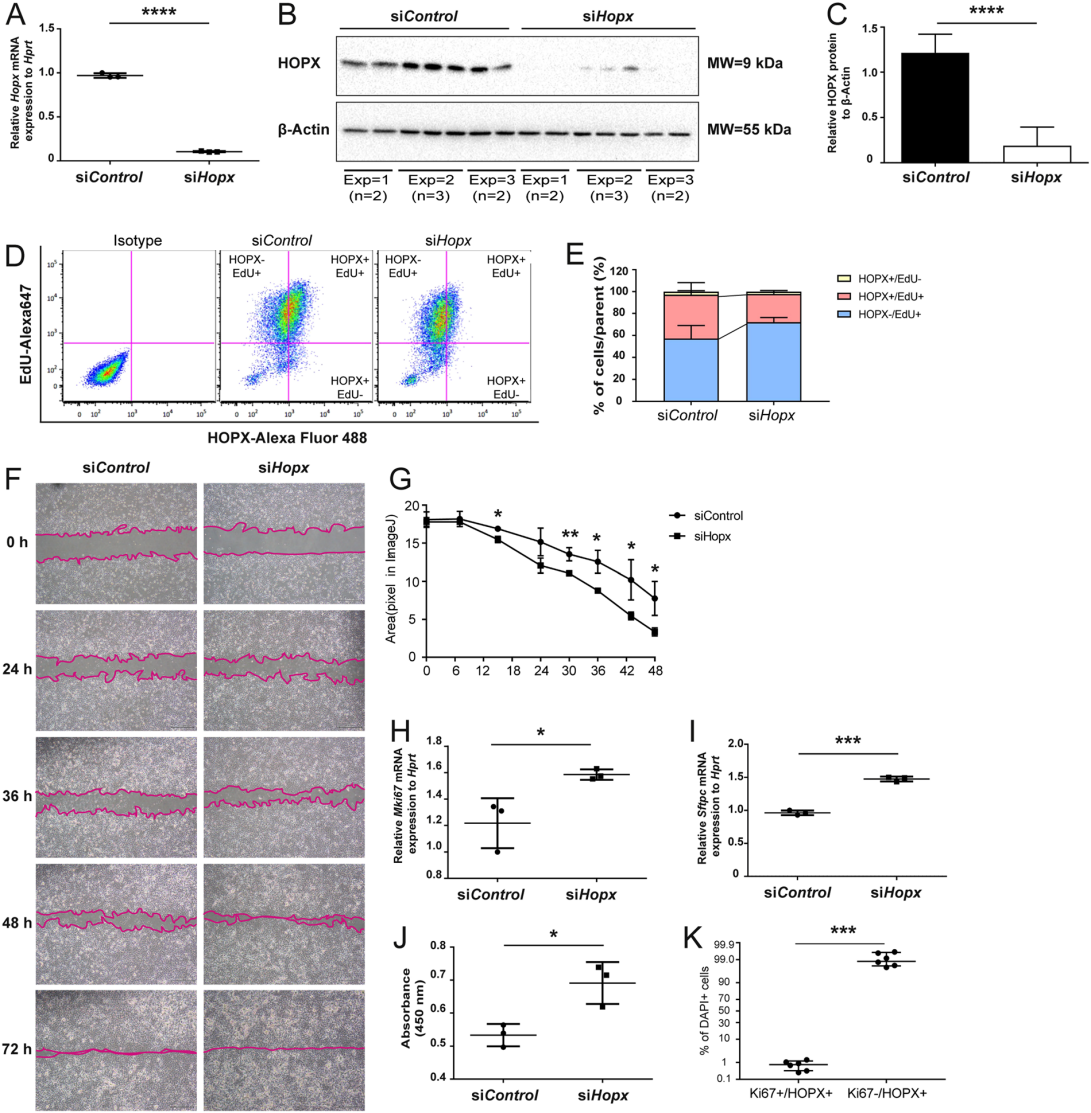
Effect of HOPX on proliferation and differentiation in MLE12 epithelial cells *in vitro*. Evaluation of si-RNA-based *Hopx* knockdown in MLE12 lung epithelial cells with (**A**) qRT-PCR of *Hopx*, (**B**) Western blotting of HOPX, (**C**) quantification of Western blotting data. (**D**) EdU assay of MLE12 lung epithelial cells co-stained with HOPX antibody *in vitro*. Alexa Fluor 647-EdU+ cells and Alexa Fluor 488-HOPX+ cells were evaluated by FCM (Representative image of n = 2). (**E**) Quantification of HOPX+ EdU+ cells and HOPX-EdU+ cells in MLE12 cells (n = 2). (**F**,**G**) Cellular scratch assay, qRT-PCR analysis of (**H**) *Mki67 and* (**I**) *Sftpc*, and (**J**) WST assay of MLE12 cells transfected with control siRNA and *Hopx* siRNA. (**J**) The ratio of Ki67 positive/negative cells with HOPX co-expression in IF. *p < 0.05, **p < 0.01, ***p < 0.005.

To investigate whether HOPX is involved in the proliferation of lung epithelial cells, we silenced *Hopx* by siRNA in the murine alveolar epithelial cell line MLE12, which endogenously expresses HOPX. We found that HOPX/*Hopx* expression was efficiently reduced in the cells as assessed by qRT-PCR (Fig. 3A) and western blotting (Fig. 3B, quantification in Fig. 3C). Next, we performed an EdU-based proliferation assay using the siRNA-transfected MLE12 cells with co-staining of HOPX by FCM. We found that *Hopx* knockdown (si*Hopx*) increased the HOPX−/EdU+ population while reducing the HOPX+/EdU+ population (Fig. 3D,E). Further, we observed an increase in proliferation using a scratch assay in si*Hopx*-transfected MLE12 cells (Fig. 3F,G). In support of these observations, we found that the knockdown of *Hopx* significantly increased *Mki67* expression as well as ATII marker *Sftpc* (Fig. 3H and I), and increased net metabolic activity as assessed by WST1 assay (Fig. 3J). In addition, we evaluated Ki67+ cells with and without HOPX in the BLM-instilled lung by IF. The number of Ki67+ cells within the HOPX+ fraction was significantly lower than that within the HOPX- fraction (Fig. 3K). Altogether, these results suggest that HOPX is involved in suppression of AEC proliferation.

### HOPX expression in IPF lungs

**Figure 4:**
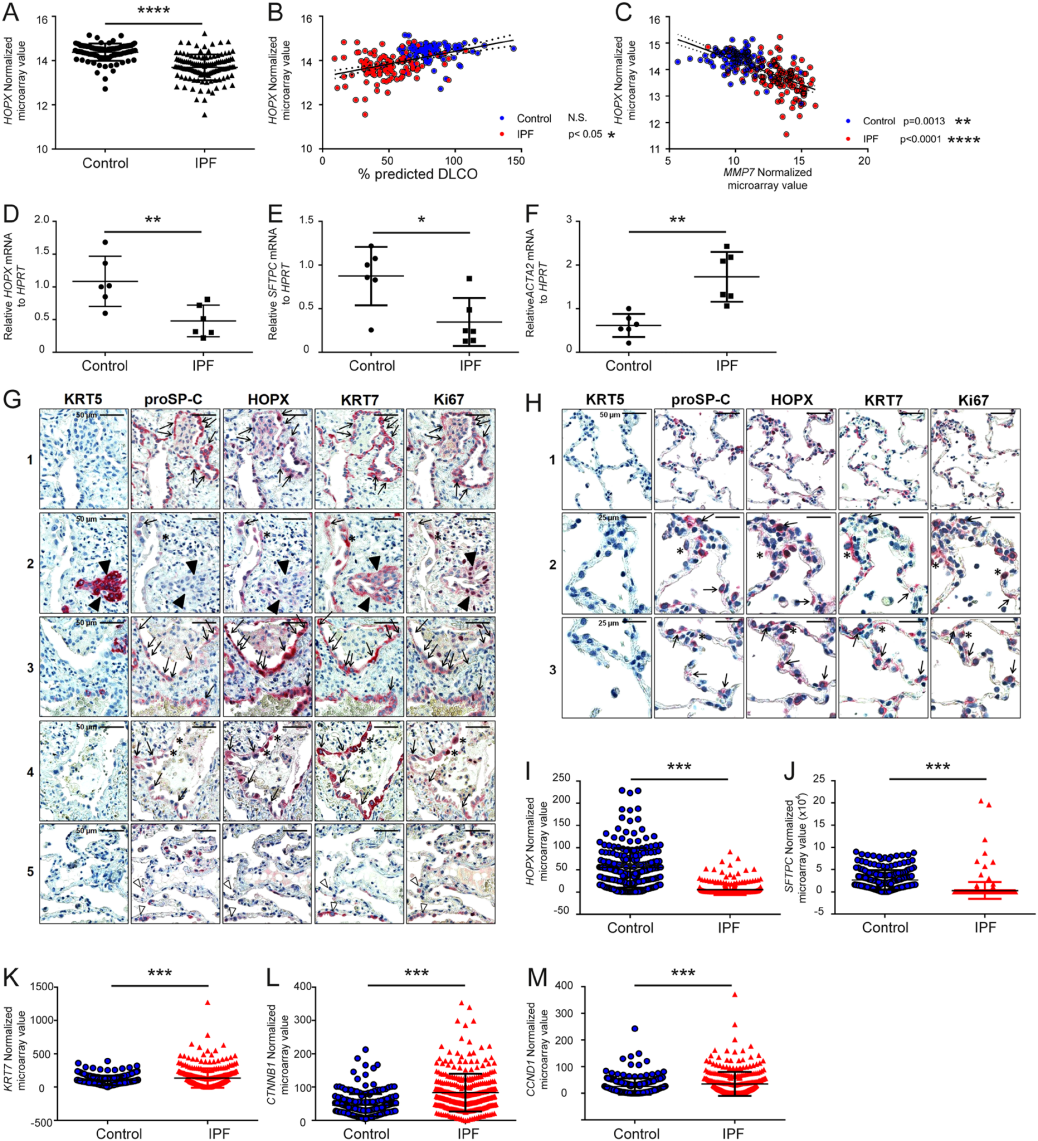
Expression and localization of HOPX in control (donor) and IPF lungs. *In silico* analysis from Lung Genomics Research Consortium (LGRC) of (**A**) *HOPX* mRNA expression in organ donor (control) and IPF lungs, (**B**) correlation between *HOPX* mRNA expression and DLco (blue dots: control, red dots: IPF). (**C**) Correlation between *HOPX* mRNA and *MMP7* mRNA in control (blue dots) and IPF (red dots) *in silico*. QRT-PCR analysis of (**D**) *HOPX* mRNA, (**E**) *SFTPC* mRNA, and (**F**) *ACTA2* mRNA from whole lung homogenate of control and IPF lungs in our cohort. (**G**) Immunohistochemical staining of serial sections of control and IPF lung tissue for KRT5, proSP-C, HOPX, KRT7, and Ki67. In IPF lungs, proSP-C^+^/KRT7^+^ cells revealed cytoplasmic or in part nuclear expression of HOPX without nuclear Ki67 (Indicated by arrows in panels 1–4). ProSP-C^low^/KRT7+ AECs of IPF-lungs also indicated robust HOPX-immunoreactivity with nuclear Ki67 expression (asterisks in panel 2 and 4). Some proSP-C+/KRT7+ cells without HOPX immunoreactivity were observed in the area of relatively preserved alveolar septa (white arrowheads in panel 5). KRT5^+^/KRT7^+^ bronchiolar basal cells did not express HOPX, and indicated nuclear expression of Ki67 (Indicated by black arrowheads in panel 2). (**H**) Immunohistochemical staining of serial sections of control donor lung tissues for KRT5, proSP-C, HOPX, KRT7, and Ki67. In donor lungs, HOPX expression was present in proSP-C+/KRT7+ ATII (arrows) and proSP-C^low^/KRT7+ AECs (asterisks) (panels 2 and 3 in Fig. 4O), and faint in normal bronchiolar epithelium. ProSP-C^low^/KRT7^+^ AECs expressing HOPX also revealed nuclear Ki67 expression (indicated by asterisks). *In silico* single cell RNA sequence analysis of (I) *HOPX*, (J) *SFTPC*, (K) *KRT7*, (L) *CTNNB1*, and (M) *CTND1* in EpCAM+/HTII280+ cells. *p < 0.05, **p < 0.01, ***p < 0.005, ****p < 0.0001.

Thus far, we have shown that HOPX contributes to adult lung injury/repair process in mouse lungs. Next, we sought to clarify whether HOPX might act as a modulator of lung injury/repair in human lungs. To this end, we first analyzed microarray datasets *in silico* to investigate the expression changes of HOPX in IPF lungs. In contrast to the mouse data, we found that *HOPX* expression was significantly decreased in whole lung homogenate from IPF lungs compared with control lungs (Fig. 4A) and the lung function parameter DL_CO_ was significantly correlated with *HOPX* expression (Fig. 4B), as indicated in the dataset from Lung Genomics Research Consortium (LGRC) (Gene Expression Omnibus Series (GSE) accession number: 47460; National Center for Biotechnology Information, Bethesda, MD, USA; https:// www. ncbi. nlm. nih. gov/ geo/). As shown in Fig. 4C by the expression of matrix metalloprotease (*MMP7*), a biomarker for progression of IPF^19^, *HOPX* expression was significantly lower in later stage than in earlier stage. Next, we confirmed decreased expression of *HOPX* (Fig. 4D) and *SFTPC* (Fig. 4E) transcripts in our own cohort (lung tissue from IPF explant and Donor lungs), while *ACTA2*, a mesenchymal marker, was increased (Fig. 4F).

Next, we stained serial lung tissue sections of organ donor and IPF patients’ lungs with cytokeratin 5 (KRT5, a marker for airway basal cells), proSP-C, HOPX, and cytokeratin 7 (KRT7, a marker for general epithelia), and Ki67, to show the expression pattern of HOPX and proSP-C as well as their proliferation status in IPF lungs (Fig. 4G and H). In IPF, KRT7+ cells are more abundant than in control lungs as previously shown in the study from our laboratory^20^. We observed that proSP-C+/KRT7+ cells of IPF lungs frequently exhibited an intense expression of HOPX without nuclear Ki67 expression (arrows in panel 1–4 in Fig. 4G). Whereas proSP-C+/KRT7+ cells without HOPX positivity were often observed within preserved alveolar septae (white arrowheads in panel 5, Fig. 4G), proSP-C+/KRT7+ cells within fibrotic regions were mostly HOPX positive (black arrows, Fig. 4G).

In addition, proSP-C^low^/KRT7+ AECs of IPF lungs also exhibited robust HOPX immunoreactivity with nuclear Ki67 expression (asterisks in panels 2 and 4 in Fig. 4G). Finally, KRT5+/KRT7+ basal cells in abnormal bronchiolar structures of IPF lungs did not express HOPX, and exhibited strong nuclear expression of Ki67 (panel 2 in Fig. 4G, indicated by black arrowheads). The data suggest that in the fibrotic area, there are HOPX^+^/proSP-C^+^/KRT7^+^ cells, HOPX^+^/proSP-C^low/−^/KRT7^+^ cells, and HOPX^−^/proSP-C^+^/KRT7^+^ cells as observed in the BLM-instilled mouse lungs. Intriguingly, HOPX^+^/proSP-C^+^/KRT7^+^ cells and HOPX^+^/proSP-C^low/−^/KRT7^+^ cells were less in the donor lungs and preserved alveolar septa, compared to fibrotic area, as observed in the BLM lungs. In addition, there are few HOPX^−^/proSP-C^+^/KRT7^+^ cells in the fibrotic area.

To further corroborate our immunohistochemistry study, we analyzed a recently published single cell RNA sequence analysis of EpCAM+/HTII280+ cells from control and IPF patients’ lungs^21^. Importantly, EpCAM+/HTII280+ cells from IPF patients exhibited decreased *HOPX* (Fig. 4I) and *SFTPC* expression (Fig. 4J), and increased expression of *KRT7* (Fig. 4K). Furthermore, *CTNNB1* (Fig. 4L) and *CCND1* (Fig. 4M) were significantly upregulated in EpCAM^+^/HTII280^+^ cells from IPF lungs. These data further support the idea that decreased HOPX expression in IPF contributes to failure of AEC regeneration and excessive/aberrant epithelial proliferation.

## Discussion

Temporal and spatial heterogeneity is one of the histological characteristics of IPF. Several stages of IPF, including initiation, progression, and the terminal stage of the disease, exist at the same time in the same lung. This heterogeneity suggests an ongoing dynamic process with different stages of alveolar injury, repair, and failed regeneration. There are various reported biomarkers of IPF^22^. Of these, serum levels of surfactant protein A (SP-A) and D (SP-D), which are synthesized and secreted by ATII, have been reported to predict mortality^23^, exacerbation^24^, or progression^25^ of IPF. The increase of these ATII-associated proteins may reflect alveolar dysfunction induced by alveolar injury processes in IPF. On the other hand, MMP7 and MMP1, which degrade extracellular matrix, are increased in plasma from IPF patients. The increase of MMPs may reflect the excessive tissue repair and remodeling^22,26^. We showed that HOPX is increased in relatively early injury and decreases along with the progression of the disease. In the present study, HOPX is shown to be upregulated in pmATII cells in BLM model of pulmonary fibrosis *in vivo*. In contrast, we found that *HOPX* mRNA was decreased in whole lung homogenate and human EpCAM+/HTII280+ cells from end-stage IPF lungs. These results may be explained by several potential areas which warrant further study. BLM lung injury is known to not fully recapitulate all aspects of clinical IPF and may represent a relatively “early” time point of regeneration in alveolar epithelium after injury whereas analyzed IPF patient samples derived from explants represent rather later disease stages. It is well-known that animals can recover and regenerate in the BLM model of fibrosis when the level of injury is not too severe. Our data allow the hypothesis that at the initial stage of regeneration, expression of HOPX is still low to allow ATII cells to undergo self-renewal, whereas at the later stage, HOPX-expressing cells are increased to suppress the aberrant/excessive proliferation and remodeling of alveoli. Thus, it is entirely plausible that a HOPX-expressing population survived the initial injury and participates in epithelial regeneration. It will be interesting in future studies to explore the levels of HOPX in different fibrotic animal models (e.g. adeno-TGF-β^27^) or models with varying degrees of severity. Importantly, we recently observed loss of HOPX in an *ex vivo* human tissue model of early IPF using precision cut lung slices^28^, and while HOPX expression increased in BLM-derived pmATII cells, human EpCAM^+^/HTII280^+^ cells in IPF lungs decreased^21^. These findings support the hypothesis that EpCAM+/HTII280+ cells in IPF have already lost their regenerative capability, while pmATII cells in BLM might still exhibit regenerative capacity in the relatively “early” repair period. Taken together, HOPX may be a marker for the extent of alveolar injury or a marker of the progression of repair, and failed regeneration.

Using the FCM-based technique and IF, we describe 3 different AEC phenotypes in terms of HOPX and proSP-C expression in murine and human alveolar epithelial cells: HOPX^−^/proSP-C^+^ ATII cells, HOPX^+^/proSP-C^−^ ATI cells, and HOPX^+^/proSP-C^+^ AECs. Of note, we observed an increase in the number of HOPX+/proSP-C+ cells in both BLM-treated mouse lungs and human IPF lungs. These HOPX^+^/proSP-C^+^ AECs could be intermediate cell types between ATII-ATI during trans-differentiation, or bipotent progenitor cells capable to differentiate either ATII or ATI cells, which have been described in the developing lung^29^ and also in alveolar epithelial progenitor lineage from adult human lung in the recent study^30^. Our data support the notion that either intermediate or bipotent progenitor cell type could emerge during alveolar injury and repair process.

HOPX is also known to be a tumor suppressor gene in several organs^31–34^. In the lung, HOPX expression inhibited lung adenocarcinoma formation and metastasis^14,15^. In our study, we demonstrate that HOPX is involved in suppression of epithelial cell proliferation *in vitro*. IHC on IPF lungs also indicated that HOPX^+^/proSP-C^+^/KRT7^+^ AECs lacked nuclear Ki67 expression. In addition, KRT5^+^/proSP-C^−^/HOPX^−^/KRT7^+^ abnormal bronchiolar basal cells in IPF showed more nuclear Ki67 expression. End-stage IPF lungs indicate fibrotic scarring as well as increased bronchiolization, i. e. enhanced bronchiolar proliferation and replacement of alveoli by bronchiolar epithelium, HOPX expression may decrease during this process along with the progression of IPF.

In IPF, alveolar epithelial regeneration is incomplete or ineffective^35^. This may be due to the persistence of “repetitive triggers” caused by genetic susceptibility^36–39^ or epigenetic changes^40,41^, resulting in permanent and persistent epithelial injury and apoptosis^42^. In a variety of studies, “maladaptive” pro-apoptotic endoplasmic reticulum stress, DNA damage as well as senescence have been well documented in the AECII of patients with sporadic and familial IPF^20,43^. These conditions might be responsible for the reduction of genes involved in alveolar maintenance, such as *HOPX*. In addition, IPF is characterized by a “hyper-responsive” regenerative loop due to re-activation of developmental pathways including sonic hedgehog^44^, TGF-β^45^, and Wnt signaling^46^, resulting in inappropriate proliferation of alveolar epithelial precursors, which are also prone to die due to replicative senescence, and presumably also due to loss of HOPX. Impaired alveolar regeneration in IPF is paralleled by the abnormal proliferation of bronchiolar basal cells, which initiate bronchiolization, leading to the migration and invasion of these bronchiolar progenitors into distal alveolar spaces, and finally replacement of alveoli. Indeed, bronchiolar basal cells which do not express HOPX, can be found in abnormal luminal position directly near ATII in alveoli^47^. Therefore, we suggest that the loss of HOPX due to increased bronchiolization of alveoli aggravate the impaired alveolar regeneration. Our data support the hypothesis that HOPX expression maintains AEC quiescence during alveolar homeostasis, whereas HOPX induction limits aberrant AEC proliferation during normal lung injury/repair. Furthermore, aberrant HOPX expression may contribute to increased alveolar senescence in IPF.

## Conclusion

HOPX contributes to alveolar injury/repair process in fibrotic lung diseases, but fails to regenerate alveolar epithelium in IPF, due to loss of HOPX. HOPX may thus be potential indicator of the progression of fibrosis.

## Methods

### Human Samples

The study protocol was approved by the Ethics Committee of the Justus-Liebig-University Giessen (No. 111/08 and 58/15), and informed consent was obtained in written form from each subject. All IPF diagnoses were made on the basis of the recent IPF consensus guidelines^48^ and a usual interstitial pneumonia (UIP) pattern was consistently observed in all used lung tissues. All methods, including those involving human samples, were performed in accordance with the relevant guidelines and regulations.

### Animals

Six to eight-week-old female wildtype C57/BL6N mice were obtained from Charles River and housed in rooms with constant humidity and temperature with 12 h light cycles and free access to water and rodent chow. All animal studies were performed under the strict governmental and international guidelines and approved by the local government for the administrative region of Upper Bavaria (Project 55.2-1-54-2532-88-12). All methods were performed in accordance with the relevant guidelines and regulations.

### Isolation and culture of primary mouse AECs

Primary mouse (pm) ATII cells were isolated as previously described with some modifications^49^. Briefly, lungs were perfused with ice-cold phosphate buffer saline (PBS) (Invitrogen, Thermo Fischer Scientific, Waltham, MA USA) via right ventricle. Then dispase (Roche Applied Science, Mannheim, Germany) and agarose (low gelling temperature) (Sigma Aldrich, St. Louis, MO, USA) were intratracheally instilled into the mouse lungs. CD45 positive white blood cells were magnetically depleted by CD45 microbeads (Miltenyi Biotec, Teterow, Germany) and pmATII cells were magnetically isolated by epithelial cell adhesion molecule (EpCAM) microbeads (Miltenyi Biotec). pmAECs were cultured on plastic plates with DMEM/F12 medium supplemented with 10% fetal bovine serum (FBS, Invitrogen, Thermo Fischer Scientific), 100 mg/l streptomycin, and 100 U/ml penicillin (Sigma Adrich) for 5 days and harvested on days 2, 3 and 5.

### Evaluation of HOPX and proSP-C expressing cells by flow cytometry

HOPX and proSP-C expression within epithelial cells was evaluated by flow cytometry (FCM). Magnetically-isolated cells were fixed and permeabilized with IntraPrep^TM^ Permeabilization Reagent (Beckman Coulter, Brea, CA, USA) and stained with HOPX mouse monoclonal antibody (sc-398703, 1:200, Santa Cruz Biotechnology, Dallas, TX, USA) and proSP-C rabbit polyclonal antibody (1:1000, Merck Millipore, Billerica, MA, USA). Normal mouse IgG (Santa Cruz Biotechnology) and rabbit serum (Life Technologies Corp.) were used for the isotype controls. Alexa Fluor 647 goat anti-mouse IgG and Alexa Fluor 488 goat anti-rabbit IgG (Life Technologies Corp.) were used for secondary antibodies. Intracellular expression of HOPX and proSP-C was detected using a FACS LSR II flow cytometer (BD Biosciences, San Jose, CA, USA). The HOPX and pro-SP-C positive populations were quantified by FlowJo software (Tomy Degital Biology Co., Ltd., Tokyo, Japan).

### HOPX expression in bleomycin-induced lung injury/repair model

Eight-week-old female wildtype C57/BL6N mice were instilled with PBS or 2 units (as a single dose of 2 U/kg body weight in 50 µl PBS) of bleomycin (BLM, Bleomycin sulfate, Almirall, Barcelona, Spain) *in vivo*. After 14 days of instillation, fixed lung tissue was used for IF. pmATII cells from PBS/BLM mouse lungs were separated as above and the cells were used for quantitative (q) RT-PCR and FCM analysis.

### Hopx siRNA transfection

Murine lung epithelial cells (MLE12) were purchased from ATCC (CRL-2110) and cultured in RPMI medium (Invitrogen, Thermo Fischer Scientific) supplemented with 10%FBS, 100 mg/l streptomycin, and 100 U/ml penicillin.MLE12 cells were grown until 70–80% confluency in six-well cluster plates and transfected with 150 pmol/l of specific double-stranded siRNA targeted against the *Hopx* transcript (sc-38672; Santa Cruz Biotechnology, Inc.) in serum-free Opti-MEM medium (Life Technologies) in combination with Lipofectamine 2000 transfection reagent (Life Technologies). Control transfections were performed using 150 pmol/l non-silencing control siRNA (SC-37007; Santa Cruz Biotechnology,Inc.). After 24 h of transfection, the cells were collected for RNA isolation, metabolic assay, FCM analysis, or lysed for western blotting.

### WST-1 metabolic assay

WST-1 metabolic assay was performed as previously described^50^.

### EdU assay

EdU-based proliferation assay was performed using Click-iT^®^ EdU Alexa Fluor^®^ 647 Imaging Kit (Invitrogen, Thermo Fischer Scientific) as manufacturer’s instructions with some modifications. Briefly, MLE12 cells were incubated with EdU solution for 24 hours, fixed with 3.7% formaldehyde, and permeabilized with 0.5% TritonX-100. Then the cells were intracellularly stained with mouse HOPX antibody as described above. Alexa Fluor 647 secondary dye was used to visualize EdU positive cells and Alexa Fluor 488 goat anti-mouse IgG was used to evaluate HOPX expression. The number of EdU positive/HOPX positive, or EdU positive/HOPX negative cells were measured and quantified by a FACS LSR II flow cytometer (BD Biosciences).

### Scratch assay

Scratch assay was performed using Culture-Insert 2 well in μ-Dish 35 mm (Nippon Genetics Co. Ltd., Tokyo, Japan) according to the manufacturer’s instructions. The gap between the cells were measured using ImageJ software (from NIH, MD, USA).

### Total RNA isolation and qRT-PCR

Total RNA from pmAECs and whole lung homogenate was isolated using Total RNA Kit, peqGOLD (VWR International, Radnor, PA, USA) according to the manufacturer’s instructions. cDNA was synthetized as previously described^28^. QRT-PCR was performed using SYBR green and the LightCycler 480 system (Roche, Mannheim, Germany). Expression changes of alveolar epithelial genes (*Hopx*, *Sftpc*, *T1α*), proliferation markers (*Mki67*, *Ctnnb1*) were evaluated by qRT-PCR. Primer sequences were as follows; m*Hopx* forward: 5′TCTCCATCCTTAGTCAGACGC3′, reverse: 5′GGGTGCTTGTTGACCTTGTT3′, m*Sftpc* forward: 5′AGCAAAGAGGTCCTGATGGA3′, reverse: 5′GAGCAGAGCCCCTACAATCA 3′, m*T1α* forward: 5′ACAGGTGCTACTGGAGGGCTT3′, reverse: 5′TCCTCTAAGGGAGGCTTCGTC3′, m*Hprt*: 5′ CCTAAGATGAGCGCAAGTTGAA3′, 5′ CCACAGGACTAGAACACCTGCTAA3′.

### Immunofluorescence staining

Immunofluorescence staining (IF) of the lung sections was performed as previously described^49^ with some modifications. Briefly, after deparaffinized, the lung sections were boiled in citrate buffer (10 mM Citric Acid, pH 6.0) for 10 minutes at 90 °C to retrieve antigens. Samples were blocked by 5% BSA with 0.1% TritonX-100 (company) in PBS for 2 hours at room temperature, and then stained with primary antibodies overnight at 4 °C. We used the following primary antibodies; HOPX mouse monoclonal antibody (1:200, Santa Cruz Inc., sc-398703) and proSP-C rabbit polyclonal antibody (1:1000, Merck Millipore, Billerica, MA, USA, AB3786). Normal mouse IgG (Santa Cruz Biotechnology) and rabbit serum (Life Technologies Corp.) were used for the isotype controls. Alexa Fluor 647 goat anti-mouse IgG and Alexa Fluor 488 goat anti-rabbit IgG (Life Technologies Corp.) were used for secondary antibodies. On the following day, the sections were washed with 1% BSA/PBS and stained with secondary antibodies for 1 hour at room temperature. Samples were visualized using a Zeiss LSM710 confocal microscope (Carl Zeiss, Oberkochen, Germany). Images were obtained, and co-localization analysis was performed with ZEN2009 software (Carl Zeiss).

### Immunohistochemistry

Immunohistochemistry (IHC) of the lung sections was performed as previously described in detail^20^. The primary antibodies used for IHC are listed as follows: proSP-C rabbit polyclonal antibody (1:750, Merck Millipore, AB3786), rabbit monoclonal antibody for human cytokeratin-5 (KRT5) (1:200, Abcam, Cambridge, United Kingdom, ab75869), rabbit monoclonal antibody for human cytokeratin-7 (KRT7) (1:200, Abcam, ab68459), HOPX mouse monoclonal antibody (1:50, Santa Cruz Inc., sc-398703), and mouse monoclonal antibody for human Ki67 (1:50, Dianova, DIA-670-P05). Immunostained lung sections were scanned with a scanning device (Nano-Zoomer, Hamamatsu), and examined histopathologically using the ‘NDP.view2 software’ at 200×, 400× and 800× original magnification. IHC for mentioned antibodies was undertaken in 12 IPF- and 5 control-donor lung samples.

### Western blotting

Western blotting was performed as previously described^20^

### *In silico* analysis

HOPX expression was evaluated using the microarray dataset from Lung Genomics Research Consortium (LGRC) and the dataset from the previous reports^21^.

### Data presentation and statistical analysis

Unless otherwise described, all data are presented as means ± standard deviation (SD). Statistical analyses were performed using the Graph Pad Prism 6 software.

For the statistical comparison of differences between two groups, the unpaired t-test was applied. For the statistical comparison of differences between three groups, one-way ANOVA with Bonferroni’s multiple comparisons test as post-test was applied. Statistical significance was defined as p < 0.05.

## Data Availability

The datasets analyzed during the current study are available from the corresponding author on reasonable request.
